# Hepatocyte growth factor and carotid intima-media thickness in relation to circulating CD34-positive cell levels

**DOI:** 10.1186/s12199-018-0705-4

**Published:** 2018-05-03

**Authors:** Yuji Shimizu, Shimpei Sato, Jun Koyamatsu, Hirotomo Yamanashi, Mako Nagayoshi, Shin-Ya Kawashiri, Keita Inoue, Shoichi Fukui, Hideaki Kondo, Seiko Nakamichi, Yasuhiro Nagata, Takahiro Maeda

**Affiliations:** 1 Department of Community Medicine, Nagasaki University Graduate School of Biomedical Sciences, Nagasaki-shi, Sakamoto 1-12-4, Nagasaki, 852-8523 Japan; 2 Department of Cardiovascular Disease Prevention, Osaka Center for Cancer and Cardiovascular Disease Prevention, Osaka, Japan; 3 Department of Island and Community Medicine, Nagasaki University Graduate School of Biomedical Sciences, Nagasaki, Japan; 4 Department of General Medicine, Nagasaki University Hospital, Nagasaki, Japan; 5 Center for Comprehensive Community Care Education, Nagasaki University Graduate School of Biomedical Sciences, Nagasaki, Japan

**Keywords:** CD34-positive cell, CIMT, Endothelial repair, HGF, Elderly men

## Abstract

**Background:**

Hepatocyte growth factor (HGF) may act as a possible biochemical index for vascular damage, although evidence for the association between HGF and carotid intima-media thickness (CIMT) is limited. Since both HGF and circulating CD34-positive cells play an important role in endothelial repair, circulating CD34-positive cell levels may influence the association between HGF and CIMT.

**Methods:**

We conducted a cross-sectional study of 269 elderly Japanese men aged 60–69 years who had undertaken an annual medical checkup from 2014 to 2015.

**Results:**

The median value for circulating CD34-positive cells was 0.93 cells/μL. Among the study population, 135 men showed low circulating CD34-positive cell levels (≤ 0.93 cells/μL). By multivariable linear regression analysis, HGF was found to be significantly positively associated with CIMT only to participants with low circulating CD34-positive cell levels, with a multi-adjusted *β* of 0.26 (*p* = 0.005) and 0.002 (0.986) for low and high circulating CD34-positive cell levels, respectively. In addition, a significant interaction was observed between HGF and circulating CD34-positive cell levels (low and high) on CIMT (multivariable *p* value of 0.049). A positive association exists between HGF and CIMT in elderly Japanese men, limited to participants with low circulating CD34-positive cell levels.

**Conclusion:**

A positive association exists between HGF and CIMT in community-dwelling elderly Japanese men, which is limited to participants with low numbers of circulating CD34-positive cells. Our findings indicate that circulating CD34-positive cell levels could determine the influence of HGF on CIMT in elderly Japanese men.

## Background

Hepatocyte growth factor (HGF) elicits beneficial effects of endothelial and tissue repair following endothelial injury since it demonstrates cytoprotective and angiogenic activity [1–3] and plays an important role in tissue regeneration [4]. Serum HGF may act as an indicator of vascular endothelial disturbance since HGF levels are elevated in participants demonstrating such a state [5, 6]. HGF also plays a crucial role in inducing endothelial progenitor cell activity such as CD34-positive cell migration and proliferation [7–9]. Since CD34-positive cells have been reported to contribute to endothelial repair [10], the status of circulating CD34-positive cells could influence the effects of HGF-mediated endothelial repair.

On the other hand, as with HGF, platelets have also been shown to play an important role in vascular endothelial repair in conjunction with circulating CD34-positive cells [11–13]. Our previous study found that platelet count is positively associated with hypertension in participants with low, but not high, circulating CD34-positive cell levels [14]. Since hypertension and endothelial dysfunction have a bidirectional relationship [14–18], circulating CD34-positive cell levels may influence the association between HGF and atherosclerosis.

To clarify these associations, we conducted a cross-sectional study of Japanese elderly men aged 60–69 years who had taken a general health checkup from 2014 to 2015.

## Methods

### Study population

The total number of male residents of Goto city aged 60–69 (estimated by the National Institute of Population and Social Security Research in March 2013) was 3264 in 2015 [19]. The study population comprised 276 male residents aged 60–69 years from the Goto Islands located in the western part of Japan, who underwent an annual medical checkup in 2014 and 2015 as recommended by the Japanese Government. Those without data for CD34-positive cells (*n* = 2), HGF (*n* = 3), IMT (*n* = 1), or blood (*n* = 1) were excluded from the study population. The remaining patients, 269 men with a mean age of 65.4 years (standard deviation (SD), 2.6; range 60–69), were enrolled in the study.

### Data collection and laboratory measurements

Trained interviewers obtained information on medical history. Current drinker (≥ 69 g/week) and current smoker were defined as drinker and smoker.

Body weight and height were measured with an automatic body composition analyzer (BF-220; Tanita, Tokyo, Japan), and body mass index (BMI; kg/m^2^) was calculated.

Systolic and diastolic blood pressures of the right arm were measured after at least 5 min of rest in a sitting position with a blood pressure measuring device (HEM-907; Omron, Kyoto, Japan) and recorded by a trained observer.

Fasting blood samples were collected in a heparin sodium tube, EDTA-2K tube, and a siliconized tube. Fresh samples (within 24 h from drawing) from the heparin sodium tube were used to determine the number of CD34-positive cells. BD (Beckton Dickinson Biosciences) Trucount™ technology, an accurate and reproducible single platform assay cited in the International Society of Hematotherapy and Graft Engineering (ISHAGE) guidelines [20] and supported by automated software on the BD FACSCant™ II system, was used to measure the number of circulating CD34-positive cells.

To measure HGF, serum samples were diluted fourfold with specific Bio-Plex sample diluents. HGF concentration was determined using a fluorescent bead-based immunosorbent assay on a suspension array. This method is recommended by the International Committee for Standardization in Hematology.

Samples from the EDTA-2K tube were used to measure white blood cell count using an automated procedure at SRL, Inc. (Tokyo, Japan). Serum triglycerides (TG), serum high-density lipoprotein cholesterol (HDLc), serum γ-glutamyltranspeptidase (γ-GTP), hemoglobin A1c (HbA_1C_), and serum creatinine were measured using standard laboratory procedures at SRL, Inc. (Tokyo, Japan). Glomerular filtration rate (GFR) was estimated by using an established method recently proposed by a working group of the Japanese Chronic Kidney Disease Initiative [21]. According to this adaptation, GFR (ml/min/1.73 m^2^) = 194 × (serum creatinine (enzyme method))^−1.094^ × (age)^−0.287^.

Measurement of carotid intima-media thickness (CIMT) by ultrasonography of the left and right carotid arteries was performed by an experienced vascular technician using a LOGIQ Book XP with a 10-MHz transducer (GE Healthcare, Milwaukee, WI, USA). Mean values for the left and right common CIMT were calculated using automated digital edge-detection software (Intimascope; MediaCross, Tokyo, Japan), with the protocol described in detail elsewhere [22]. Intimascope is an innovative software developed for CIMT measurement to minimize measurement errors. This software makes it possible to recognize automatically the edges of the internal and external membranes of the blood vessels and also to determine automatically the distance at a sub-pixel level (estimated to be 0.01 mm) by using a three-dimensional polynomial measurement formula [23]. The reproducibility of CIMT measurements by means of intimascope for our part of the study population (*n* = 25) was shown to be satisfactory: the respective intra-observer variations for CIMT assessed by two examiners were simple correlation coefficients (*r*) = 0.98 (*p* < 0.001) and *r* = 0.97 (*p* < 0.001), and the inter-observer variation was *r* = 0.80 (*p* < 0.001).

### Statistical analysis

Characteristics of the study population in relation to circulating CD34-positive cell levels were expressed as mean ± standard deviation except for TG, γ-GTP, and HGF. Since these three factors showed a skewed distribution, the characteristics of the study population were expressed as median [the first quartile, the third quartile], followed by logarithmic transformation. The regression model for mean values was used for calculating *p* values.

A simple correlation analysis and multiple linear regression analysis of CIMT were conducted with relevant factors adjusted for confounding factors based on circulating CD34-positive cell levels at a median value (0.93 cells/μL). Alcohol consumption and smoking status are well-known factors that affect vascular remodeling. Since γ-GTP is recognized as a factor that is influenced by alcohol consumption, and WBC as a factor that is influenced by smoking status [24], we added γ-GTP and WBC as the confounding factors to the present analysis instead of using alcohol consumption and smoking status directly as was done in a previous study of ours [25]. For the multiple linear regression analysis, adjustments were made for age, systolic blood pressure (mmHg), BMI (kg/m^2^), TG (mg/dL), HDLc (mg/dL), γ-GTP (IU/L), HbA1c (%), GFR (mL/min/1.73m^2^), and WBC (cells/μL).

Since bone marrow-derived endothelial progenitor cells such as CD34-positive cells have been reported to play an important role in maintaining the vascular endothelium [26, 27], and the level of circulating CD34-positive cells may serve as a direct indicator of vascular maintenance activity [14, 15, 28, 29], we also evaluated the association between HGF and CIMT, stratified by circulating CD34-positive cell levels [low (< 0.93 cells/μL) and high (≥ 0.93 cells/μL)].

To evaluate the impact of HGF levels [median value (301.0 pg/mL)] and circulating CD34-positive cell levels [median value (0.93 cells/μL)] on CIMT, we also created and investigated relevant crosstabs.

All statistical analyses were performed with SAS system for Windows (version 9.4: SAS Inc., Cary, NC). Values of *p* < 0.05 were regarded as being statistically significant.

## Results

Among the study population, 135 participants were categorized as having low CD34-positive cell levels (≤ 0.93 cells/μL), and 134 participants were categorized as having high levels (> 0.93 cells/μL).

### Characteristics of the study population

Characteristics of the study population based on circulating CD34-positive cell levels are shown in Table 1. Participants with high CD34-positive cell levels show significantly higher values for BMI, TG, and WBC.

**Table 1:** Characteristics of the study population based on CD34-positive cell levels

	Low CD34-positive cells (≤ 0.93 cells/μL)	High CD34-positive cells (> 0.93 cells/μL)	*p*
No. of participants	135	134	
Age, years	65.5 ± 2.6	65.2 ± 2.6	0.378
Systolic blood pressure, mmHg	136 ± 17	137 ± 18	0.665
Diastolic blood pressure, mmHg	84 ± 12	85 ± 11	0.513
Drinker, %	23.0	23.1	0.974
Smoker, %	16.3	16.4	0.979
Body mass index (BMI), kg/m^2^	23.1 ± 3.0	24.0 ± 2.8	0.014
Serum triglycerides (TG), mg/dL	89 [66–111]^a^	110 [72–138]^a^	0.028^b^
Serum HDL-cholesterol (HDLc), mg/dL	57 ± 14	57 ± 14	0.744
Serum γ-glutamyltranspeptidase (γ-GTP), IU/L	31 [20–58]^a^	35 [23–50]^a^	0.379^b^
Hemoglobin A1c (HbA1c), %	5.6 ± 0.7	5.8 ± 0.7	0.168
Serum creatinine, mg/dL	0.82 ± 0.14	0.85 ± 0.15	0.065
Glomerular filtration rate (GFR), mL/min/1.73m^2^	75.0 ± 14.4	71.8 ± 12.6	0.054
Hepatocyte growth factor (HGF), pg/mL	285.6 [215.8–416.9]^a^	308.1 [231.6–429.2]^a^	0.325^b^
White blood cell count (WBC), cells/μL	5325 ± 1460	6217 ± 1348	< 0.001
Carotid intima-media thickness (CIMT), mm	0.70 ± 0.12	0.69 ± 0.13	0.881

Values are mean ± standard deviation

^a^Values are median [the first quartile, the third quartile]. Regression model for mean values was used for determining *p* values

^b^Logarithmic transformation was used for evaluating *p*

### Association between HGF and CIMT in relation to circulating CD34-positive cell levels

**Fig. 1:**
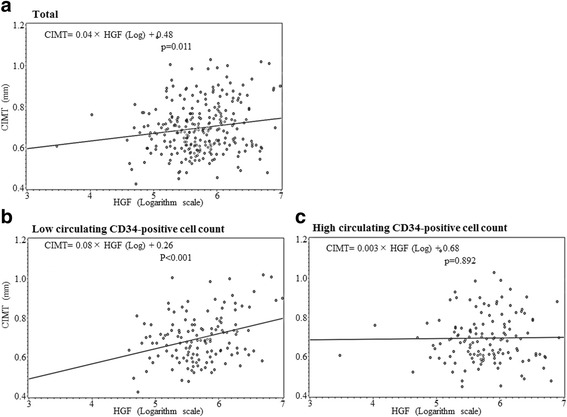
Scatter plot of hepatocyte growth factor (HGF) and carotid intima-media thickness (CIMT) for participants with **a** total, **b** low circulating CD34-positive cells (≤ 0.93 cells/μL), and **c** high circulating CD34-positive cells (> 0.93 cells/μL)

Table 2 shows a simple correlation analysis of CIMT and other variables. For the total participants, HGF was significantly positively associated with CIMT. This association was limited to participants with low CD34-positive cell levels, with essentially the same associations seen from a simple linear regression analysis (Fig. 1). After further adjustment for possible confounding factors, for total participants even the association became non-significant value, for participants with low and high CD34-positive cell levels, these associations remained unchanged (Table 3).

**Table 2:** Simple correlation analysis of carotid intima-media thickness (CIMT) and other variables

	Total subjects		Low CD34-positive cells (≤ 0.93 cells/μL)		High CD34-positive cells (> 0.93 cells/μL)	
	*r*	*p*	*r*	*p*	*r*	*p*
No. of participants	269		135		134	
Age	0.17	0.005	0.13	0.141	0.21	0.013
Systolic blood pressure	0.14	0.023	0.17	0.047	0.11	0.212
Body mass index (BMI)	0.01	0.810	0.05	0.592	− 0.01	0.864
Serum triglycerides (TG)	− 0.01	0.874	0.01	0.865	− 0.03	0.752
Serum HDL-cholesterol (HDLc)	− 0.03	0.593	− 0.13	0.125	0.06	0.480
γ-Glutamyltranspeptidase (γ-GTP)	0.04	0.517	− 0.03	0.691	0.12	0.179
Hemoglobin A1c (HbA1c)	− 0.003	0.966	0.02	0.781	− 0.03	0.758
Glomerular filtration rate (GFR)	0.07	0.255	0.10	0.243	0.03	0.697
White blood cell count (WBC)	0.11	0.086	0.17	0.051	0.05	0.525
Hepatocyte growth factor (HGF)	0.15	0.011	0.31	< 0.001	0.01	0.892

TG, γ-GTP, and HGF are calculated in logarithm values

**Table 3:** Multiple linear regression analysis of carotid intima-media thickness (CIMT)

	Total subjects			Low CD34-positive cells (≤ 0.93 cells/μL)			High CD34-positive cells (> 0.93 cells/μL)		
	B	β	*p*	B	β	*p*	B	β	*p*
No. of participants	269			135			134		
Age	0.01	0.16	0.007	0.004	0.08	0.344	0.01	0.23	0.013
Systolic blood pressure	0.0009	0.12	0.047	0.001	0.17	0.072	0.0005	0.07	0.449
Body mass index (BMI)	− 0.0006	− 0.01	0.829	0.001	0.03	0.762	− 0.0009	− 0.02	0.844
Serum triglycerides (TG)	− 0.0004	− 0.05	0.528	− 0.001	− 0.07	0.503	0.0002	0.03	0.804
Serum HDL-cholesterol (HDLc)	− 0.02	− 0.07	0.361	− 0.02	− 0.06	0.574	− 0.01	− 0.06	0.555
γ-Glutamyltranspeptidase (γ-GTP)	0.01	0.03	0.669	− 0.02	− 0.09	0.367	0.02	0.11	0.231
Hemoglobin A1c (HbA1c)	− 0.01	− 0.04	0.517	− 0.02	− 0.08	0.363	− 0.01	− 0.05	0.617
Glomerular filtration rate (GFR)	0.0005	0.06	0.342	0.001	0.06	0.490	0.0003	0.03	0.749
White blood cell count (WBC)	0.00001	0.10	0.145	0.00001	0.07	0.475	0.00001	0.13	0.194
Hepatocyte growth factor (HGF)	0.03	0.12	0.070	0.06	0.26	0.005	0.0004	0.002	0.986

All listed variables have been entered in multiple linear regression analysis. TG, γ-GTP, and HGF are calculated in logarithm values

B parameter estimate, β standardized parameter estimate

### Effects of modification of circulating CD34-positive cell levels on the association between HGF and CIMT

As the positive association between HGF and CIMT was observed only in participants with low circulating CD34-positive cell levels, we tested the effect of differing CD34-positive cell levels (low and high) on the slope of HGF and CIMT and found significant values using both a simple (*p* = 0.011) and multivariable linear model (*p* = 0.049).

### Mean values of carotid intima-media thickness by HGF levels and CD34-positive cell levels

Among the participants with lower circulating CD34-positive cell levels than those of the reference group with low HGF levels, significantly higher values for CIMT were observed for participants with high HGF levels, but not for those with high circulating CD34-positive cell levels. No significant differences in CIMT between participants with high and low circulating CD34-positive cell levels were observed for participants with either high or low HGF levels (Table 4).

**Table 4:** Mean values of carotid intima-media thickness (CIMT) in terms of hepatocyte growth factor (HGF) levels and CD34-positive cell levels

	Low CD34-positive cells (≤ 0.93 cells/μL)	High CD34-positive cells (> 0.93 cells/μL)	*p*
Low HGF (≤ 301.0 pg/mL)	0.67 ± 0.11	0.69 ± 0.13	0.319
High HGF (> 301.0 pg/mL)	0.73 ± 0.13	0.70 ± 0.13	0.201
*p*	0.004	0.586	

## Discussion

The major finding of the present study is a significant positive association between HGF and CIMT in community-dwelling elderly Japanese men aged 60–69 years, limited to participants with low circulating CD34-positive cells.

A previous study of 317 community participants aged over 50 reported that those with higher levels of HGF show significantly higher CIMT values than those with lower values (8.2 ± 1.2 mm and 7.8 ± 1.4 mm, *p* < 0.01, respectively) [30]. Our results showing a significant positive association between HGF and CIMT by simple correlation analysis in total participants are compatible with the aforementioned study.

We also found further evidence that this positive association is limited to participants with low circulating CD34-positive cell levels.

Serum HGF levels were elevated in participants with a disturbance in vascular endothelial cells, indicating that HGF could act as an indicator of vascular endothelial disturbance [5, 6]. Since HGF has both cytoprotective and angiogenic activities [1–3] and plays an important role in tissue regeneration [4], HGF demonstrates beneficial effects of endothelial and tissue repair following vascular endothelial injury.

In addition, in the bone marrow microenvironment, HGF is known as a polyfunctional cytokine that is produced by human bone marrow stromal cells and directory or indirectly promotes proliferation, adhesion, and survival of human CD34-positive cells [9]. CD34-positive cells have been reported to contribute to endothelial repair [10, 14], and HGF promotes endothelial cell differentiation and increases endothelial progenitor cell migration and proliferation [7, 8]. Both HGF and CD34-positive cells may be positively associated with vascular damage. In fact, these two factors are observed in human atherosclerotic lesions [31–33].

However, increased numbers of circulating CD34-positive cells are associated with a decrease in the extent of subclinical atherosclerosis in asymptomatic men [34], while the number of total risk factors for carotid atherosclerosis with high levels of HGF is significantly greater compared to low HGF among community-dwelling participants [30]. This paradoxical phenomenon between HGF and circulating CD34-positive cell count on atherosclerosis may result from a consumptive reduction of circulating CD34-positive cells. When the endothelium sustains damage, both HGF production and circulating CD34-positive cells become elevated. However, if the endothelial disturbance is severe, a large proportion of CD34-positive cells become mature cells (CD34-negative cells) by differentiating into foam cells and endothelial cells [11], resulting in a low level of circulating CD34-positive cells remaining. Therefore, high HGF with low levels of circulating CD34-positive cells may indicate the presence of aggressive endothelial repair leading to atherosclerosis, while higher HGF along with high levels of circulating CD34-positive cells may indicate the presence of sufficient endothelial repair since no evidence of consumptive reduction of CD34-positive cells is observed. Furthermore, our crosstab analysis showed no statistically significant differences in CIMT values between high and low circulating CD34-positive cell levels, whether in participants with low or high HGF levels. These findings also support our hypothesis that increased productivity and consumptive reduction may determine the circulating CD34-positive cell count for subjects with active vascular remodeling. This hypothesis is based on the notion that because consumptive reduction of CD34-positive cells followed by increased production of these cells might constitute a strong confounding factor for the association between CIMT and circulating CD34-positive cells among participants with high HGF levels, while no aggressive endothelial repair that would affect the progression of CIMT is necessary for those with low HGF levels.

Circulating CD34-positive cells have also been revealed to play an important role in vascular endothelial repair in conjunction with platelets [11–13], and platelets are significantly positively associated with hypertension in participants with low, but not high, circulating CD34-positive cell levels [14]. Our present results are supported by these studies since a vicious cycle exists between hypertension and endothelial dysfunction [14–18], and the different associations between circulating CD34-positive cells and platelets and hypertension [14] could be induced by consumptive reduction of the former.

Although the sample used for our study was small, it is the largest sample used to date for a study dealing with circulating CD34-positive cell levels in a general elderly population selected with strict criteria, since the participants were restricted to men in a narrow age range because differences in gender and age can act as strong confounding factors on associations between CD34-positive cells, HGF, and CIMT [25, 35–40]. For this reason, even age was slightly but significantly positively associated with CIMT (simple correlation coefficient (*r*) = 0.17 *p* < 0.005) in our study, while no such associations were observed for logarithm-transformed CD34-positive cells (*r* = − 0.006, *p* = 0.034) and HGF (*r* = 0.03, *p* = 0.646).

Potential limitations of this study warrant consideration. Although the level of circulating CD34-positive cells influenced on the association between HGF and CIMT significantly, no data was available with regard to the evaluation of endothelial function. Further analyses that include endothelial function-related data such as flow-mediated dilation (FMD) will be necessary. Additionally, because this was a cross-sectional study, causal relationships were not able to be established.

## Conclusions

In conclusion, a positive association exists between HGF and CIMT in community-dwelling elderly Japanese men, which is limited to participants with low numbers of circulating CD34-positive cells. Our findings indicate that circulating CD34-positive cell levels could determine the influence of HGF on CIMT in elderly Japanese men and can be expected to serve as an effective tool for the clarification of the roles played by HGF and CD34-positive cells in the progression of atherosclerosis.

## Abbreviations

BD: Beckton Dickinson Biosciences
BMI: Body mass index
CIMT: Carotid intima-media thickness
GFR: Glomerular filtration rate
HbA1c: Hemoglobin A1c
HDLc: High-density lipoprotein cholesterol
HGF: Hepatocyte growth factor
ISHAGE: International Society of Hematotherapy and Graft Engineering
TG: Triglycerides
γ-GTP: γ-Glutamyltranspeptidase
